# Perspective: The Benefits of Including Flavored Milk in Healthy Dietary Patterns

**DOI:** 10.1016/j.advnut.2023.06.002

**Published:** 2023-06-07

**Authors:** Kristin Ricklefs-Johnson, Matthew A. Pikosky

**Affiliations:** National Dairy Council, Rosemont, IL, United States

**Keywords:** milk, flavored milk, childhood, adolescence, diet quality, metabolic health

## Abstract

The Dietary Guidelines for Americans recommend two-and-a-half cup equivalents of low-fat and fat-free dairy foods per day for children 4–8 y and 3 cup equivalents per day for adolescents aged 9–18 and adults. Currently, the Dietary Guidelines for Americans recognizes 4 nutrients as being of public concern because of suboptimal levels in the diet. These include calcium, dietary fiber, potassium, and vitamin D. In the American diet, dairy foods are leading contributors of calcium, vitamin D, and potassium. Milk, because of its unique nutrient package that provides shortfall nutrients to the diets of children and adolescents, remains an underpinning of dietary recommendations and is included with school meals. Despite this, milk consumption is declining, and >80% of Americans do not meet recommendations for dairy. Data indicate that children and adolescents who consume flavored milk are more likely to consume more dairy and adhere to healthier overall dietary patterns. Flavored milk, however, receives more scrutiny than plain milk because of its contribution of added sugar and calories to the diet and concerns over childhood obesity. Therefore, the purpose of this narrative review is to describe trends in beverage consumption in children and adolescents aged 5–18 y and highlight the science that has examined the impact of including flavored milk in overall healthy dietary patterns within this population.

**Statement of Significance**

Recent proposed changes to school meal guidelines have questioned the importance of flavored milk in the eating patterns of children and adolescents and its contribution to key nutrients including added sugar and saturated fat. Therefore, it is important to examine the current literature surrounding this topic to determine if the data support the removal of flavored milk as part of an overall healthy diet pattern. This review aims to better definethe role of flavored milk in achieving nutritional adequacy, potential impact on chronic disease risks, and key areas of research that should be considered in future studies.

## Introduction

The Dietary Guidelines for Americans (DGA) 2015–2020 and 2020–2025 classified vitamin D, calcium, potassium, and fiber as nutrients of public health concern because they are underconsumed by the entire population [1,2]. Underconsumption of these nutrients was linked with adverse health outcomes, including clinically relevant deficiencies and increased risks of osteoporosis and cardiovascular disease in adulthood [2]. As such, it is important to encourage the consumption of foods and dietary patterns that support sufficient intake of these nutrients (among others). Meanwhile, the consumption of milk, a nutrient-dense and cost-effective source of 13 essential nutrients, including 3 of the 4 nutrients of concern identified by the DGA, calcium, potassium, and vitamin D, has been steadily declining over the last 7 decades [3]. In fact, >80% of the population does not meet the DGA’s dairy recommendations [1].

Data indicate that children and adolescents who consume flavored milk are more likely to consume dairy foods [4] and adhere to healthier overall dietary patterns [5,6]. However, flavored milk is often scrutinized over its contribution to added sugars and calories to the diet. Thus, debate still exists about whether flavored milk should be included as part of healthy dietary recommendations.

The purpose of this narrative review is to describe trends in beverage consumption in United States children and adolescents ages 5–18 y and highlight the available literature examining the impact of flavored milk in their diet, with a focus on nutrient intake/adequacy, body weight, and body composition.

## Beverage Consumption Trends and Preferences among Children and Adolescents

The consumption of fluid milk in the United States has been steadily declining for decades, including in children and adolescents [3]. In 1970, United States per capita consumption of fluid milk was 0.96 cups per person per day; in 2019 it was 0.49 cups per person per day [3]. Interestingly, NHANES data from the past 2 decades show an overall decrease in total beverage intake including soft drinks, <100% fruit juice, fruit drinks, and other sugar-sweetened beverages (SSBs). However, data do show that as children age, SSBs, including soft drinks, displace cow’s milk in the diet [7]. Data from several studies indicated that children preferred flavored milk to plain milk and reported that consumption of flavored milk increased as consumption of plain milk decreased (Table 1) [[8], [9], [10], [11]]. A cross-sectional analysis of data obtained from 3229 children aged 6–11 y that were part of 3 nationally representative surveys of United States school-aged children observed an increase from 19% to 39% per capita consumption of flavored whole-milk [11]. This increase amounted to an increase from 27 to 63 kcal/d and was observed alongside a decrease in total per capita milk consumption from 218 to 170 kcal/d and total per capita “high-fat, low-sugar milk” consumption from 168 to 86 kcal/d [11]. Another study that assessed beverage consumption trends among United States children utilizing cross-sectional data from NHANES 1976–1980, 1988–1994, and 2001–2006 observed a 14% increase in flavored milk consumption from the first to the third NHANES survey among 3398 children aged <1–5 y [9]. In an intervention trial conducted in elementary schools in Canada, researchers reported a 12.3% decrease in total milk consumption among 1205 children and adolescents in grades 1–8 when chocolate milk was removed from schools [10]. Researchers observed that several factors drove milk consumption behaviors; whereas location and cost impacted total milk consumption, the preference for flavored milk and taste were also major contributors [10]. The results from these studies indicated a preference for flavored milk among children and adolescents, with taste as a driver for consumption. They also indicated that removal of flavored milk as an option for children and adolescents in school lunch programs was associated with less milk consumption overall that may have a negative impact on overall intakes of certain nutrients including vitamin A, calcium, phosphorus, magnesium, and potassium [5,11,12].

**TABLE 1:** Studies assessing the role of flavored milk in child and adolescent beverage consumption trends and preferences

Reference	Objective(s)	Design	Sample	Relevant results	Cost of flavored milk consumption	Benefit of flavored milk consumption
Lasater et al. 2011 [11]	Investigate beverage patterns and trends among United States school-aged children	Cross-sectional analyses of national survey data from United States school-aged children	*n* = 3 nationally representative surveys involving 3229 children 6–11 y of age	Total per capita calories from SSB increased from 1989 to 2008 (130–212 kcal/d, *P* < 0.05); within SSBs, largest increases per capita were fruit drinks and soft drinks (90–118 kcal/d, *P* < 0.05); high-fat, high-sugar milk (28–63 kcal/d, *P* < 0.05), and sports drinks (1–9 kcal/d, *P* < 0.05). Total per capita calories from high-fat, low-sugar milk contributed to a decrease in caloric nutritional beverage per capita trends (168–86 kcal/d, *P* < 0.05). Percent consumption from 1989 to 2008 increases: fruit drinks and soft drinks: 67%–77%; high-fat, high-sugar milk: 19%–39% (*P* < 0.05). Milk consumption trends: total per capita milk intake decreased from 218 to 170 kcal/d (*P* < 0.05); high-fat, low-sugar milk intake decreased from 168 to 86 kcal/d (*P* < 0.05)	ND	ND
Fulgoni and Quann, 2012 [9]	Assess beverage consumption trends in children	Cross-sectional analyses of 1976–1980, 1988–1994, and 2001–2006 NHANES	*n* = 3398 American children <1–5 y of age	Flavored milk consumption increased to 14% and fruit juice increased to more than 50% of the population from first to third NHANES survey (*P* < 0.001). Milk was consistently the largest contributor of calories, calcium, phosphorus, magnesium, and potassium to the American diet	Contributes more energy than other beverages to the American Diet	Contributes more micronutrients than other beverages to the American diet
De Pelsmaeker et al. 2013 [8]	Investigate the consumption of milk and flavored milk	Cross-sectional analysis utilizing questionnaires and taste sampling	*n* = 513 Belgian children 8–13 y of age	Children preferred and consumed more flavored than white milk (*P* < 0.001) and indicated taste was more important than health (*P* < 0.001)	ND	Better taste compared with white milk Higher consumption compared with white milk
Henry et al. 2015 [10]	Measure milk consumption (plain and flavored) by children in an elementary school environment and investigate factors contributing to milk choice	Intervention trial	*n* = 1205 Canadian children and adolescents in grades 1–8	Total milk intake decreased by 12.3% when chocolate milk was removed from schools (*P* < 0.01). Total milk intake was associated with location (*P* = 0.035) and cost (*P* = 0.001)	ND	Increased milk consumption when offered in addition to white milk at school

Abbreviation: SSB, sugar-sweetened beverage.

## Contributions of Flavored Milk to Nutrient Status and Diet Quality among Children and Adolescents

In the United States, it is estimated that >80% of the population have dietary patterns that are low in vegetables, fruits, and dairy foods [1]. In fact, the DGA, 2020–2025 identified 4 nutrients of public health concern for underconsumption among Americans: dietary fiber, vitamin D, calcium, and potassium [1]. The DGA 2020–2025 are intended to promote the intake of a variety of food groups, including dairy, to promote both optimal nutrient status and health outcomes as well as prevent chronic disease across all stages of life. It recommends 2.5 servings of dairy products for children 4–8 y of age and 3 servings for those 9–18 y of age to help ensure they meet nutrient intake recommendations, promote healthy growth and development, as well as reduce risk of chronic diseases [1]. A body of literature consisting of cross-sectional analyses of children and adolescents conducted over the last 15 y indicated that flavored milk consumption contributed to higher micronutrient intakes compared with those who did not consume flavored milk [5,6,[12], [13], [14], [15], [16]] supporting a role for flavored milk in helping children and adolescents meet nutrition recommendations (Table 2). A cross-sectional analysis of NHANES 2011–2014 indicated that milk and flavored milk were leading food sources of calcium, vitamin D, and potassium—3 nutrients of public health concern—in the diets of in American children [16]. Dairy products make a significant contribution to the United States population’s nutrient intakes. Children 2 y of age and older who meet dairy recommendations are less likely to be below recommendations for several essential nutrients including calcium, magnesium, phosphorus, protein, riboflavin, vitamin A, vitamin B12, vitamin D, selenium, potassium, and choline [17]. Furthermore, milk provides, on average, over 35% of the daily vitamin D, 19% of the daily calcium, and 9% of the daily potassium intake of Americans 2 y and older, [17] and for calcium, potassium, and vitamin D, milk was the top-ranked food source in all age groups [16]. Lower milk and dairy intake make it more difficult for children and adolescents to meet DGA for dairy and nutrient intake recommendations resulting in negative impacts on development because milk and the nutrients it provides are associated with growth and bone health. The DGA also identified food components to limit, such as added sugars, saturated fat, and sodium [1]. To date, flavored milk has not been identified as a primary source of those nutrients to limit in children and adolescents. In addition, the current DGA acknowledges that a small amount of added sugars can be added to nutrient-dense foods and beverages to improve palatability and help meet food group recommendations [1]. In line with these statements, the American Academy of Pediatrics states “flavored milk provides a good example of the balance needed to limit added sugars yet promote nutrient rich foods” [18].

**TABLE 2:** Studies assessing the role of flavored milk in child and adolescent nutrient status and diet quality

Reference	Objective(s)	Design	Sample	Relevant results	Cost of flavored milk consumption	Benefit of flavored milk consumption
Murphy et al. 2008 [5]	Compare nutrients intakes and body measure among children and adolescents drinking flavored milk (with or without plain milk), exclusively plain milk, and no milk	Cross-sectional analysis of NHANES 1999–2002	*n =* 7557 children and adolescents 2–18 y of age	Flavored milk intake was associated with higher total milk intake (*P <* 0.05). Vitamin A, calcium, phosphorus, magnesium, potassium, and saturated fat (adjusted for energy intake and age) were comparable among milk drinking groups; higher than milk nondrinkers (*P <* 0.05). Added sugars intake did not differ between flavored milk drinkers and milk nondrinkers. BMI of milk drinkers was comparable with or lower than milk nondrinkers (*P <* 0.05)	ND	Higher nutrient intake and comparable added sugars and BMI than milk nondrinkers
Fulgoni and Quann, 2012 [9]	Assess beverage consumption trends in children	Cross-sectional analyses of 1976–1980, 1988–1994, and 2001–2006 NHANES	*n =* 3398 American children <1–5 y of age	Flavored milk consumption increased to 14% and fruit juice increased to more than 50% of the population from first to third NHANES survey (*P <* 0.001). Milk was consistently the largest contributor of calories, calcium, phosphorus, magnesium, and potassium to the American diet	Contributes more energy than other beverages to the American Diet	Contributes more micronutrients than other beverages to the American diet
Fayet et al. 2013 [12]	Determine associations among milk drinking (plain or flavored) and milk and nutrient intakes in children	Cross-sectional analysis of 2007 Australian National Children’s Nutrition and Physical Activity Survey	*n =* 4487 Australian children and adolescents 2–16 y of age	Milk drinking (plain or flavored) was associated with higher total milk, calcium, phosphorus, magnesium, potassium, and iodine intake in and were more likely to meet the EAR for calcium in comparison with milk nondrinkers (*P <* 0.05). Flavored milk drinkers were more likely to meet the EAR for calcium than exclusive plain milk drinkers, with 9–16-y olds being 1.7 times more likely to meet the EAR for calcium than exclusive plain milk drinkers (*P <* 0.01). Flavored milk drinkers had higher intakes of total sugar and energy than exclusive plain milk drinkers (*P <* 0.05). NS difference between BMI, WC or PA levels between flavored and exclusive plain milk drinkers	Higher total sugar and energy intake	Higher micronutrient intake compared with white milk drinkers and milk nondrinkers Greater likelihood of meeting calcium recommendations compared with white milk drinkers and milk nondrinkers No impact on body weight or composition despite greater sugar and energy intake than white milk drinkers
Nicklas et al. 2013 [15]	Assess the contribution of flavored and white milk to the diets of American children and adolescents	Cross-sectional analysis of NHANE 2003–2006	*n =* 7332 children and adolescents 2–18 y of age	Flavored and white milk contributed, respectively, 2%–6% total energy; 3%–12% saturated fats; 1%–3% sodium; and 4%–0% added sugars. White milk contributed 21% vitamin A; 54% vitamin D; 29% calcium; 17% potassium; 12% magnesium; and 19% phosphorus, which exceeded 10% of total intake. Flavored milk contributed 5% vitamin A; 11% vitamin D; 6% calcium; 4% potassium; 3% magnesium; and 4% phosphorus	ND	Contributed nutrients to the diet
Nicklas et al. 2017 [6]	Assess the contribution of flavored milk to the diets of American children and adolescents	Cross-sectional analysis of NHANES 2001–2012	*n =* 20,329 American children and adolescents 2–18 y of age	3564 participants were flavored milk consumers. Flavored milk consumers consumed more milk; ages 2–3 had higher SFA intake; and ages 14–18 had higher percent energy from SFA than flavored milk nonconumers. Flavored milk consumers had a lower mean percentage meeting AI for fiber and higher percentage meeting the EAR for calcium. Consumers 4–8 and 9–13 had higher mean percentage meeting the EAR for magnesium	Higher SFA and percent energy from SFA intake and lower fiber intake than flavored milk nonconsumers	Higher calcium and magnesium intake than flavored milk nonconsumers
Lepicard et al. 2017 [14]	Analyze the nutritional quality of children’s breakfasts in France	Cross-sectional analysis	*n =* 529 French children 9–11 y of age	Breakfast provided 22.9% of recommended daily energy with the most frequently consumed food being flavored milk (50.5%). 83% of breakfast consumers consumed flavored milk. When adjusted for energy, the foods contributing most to total nutrient intake were RTEC, milk, flavored milk, and fruits	ND	Contributed nutrients to the diet
Romo-Palafox et al. 2018 [19]	Ascertain the relationship between beverage selection and dietary quality of packed lunches for preschool children using the HEI-2010	Cross-sectional analysis	*n =* 607 foods packed by parents	Milk and flavored milk made up 14% and 3.7%, respectively, of beverages packed by parents. Lunches with plain milk had the highest HEI scores (59.3) followed by lunches with 100% fruit juice (56.9) and flavored milk (53.2)	ND	Indicator of diet quality
O’Neil et al. 2018 [16]	Determine the food sources of energy, nutrients of public health concern, and nutrients to limit with a focus on dairy foods	Cross-sectional analysis of NHANES 2011–2014	*n =* 5876 children and adolescents 2–18 y of age	Milk was the first, second, and fourth leading source of energy in children 2–5; 6–11, and 12–18 y of age (8.9%; 6%; and 5.7%), respectively. Milk and flavored milk were the first and third leading source of calcium in children 2–5 y (36.2% and 7.6%) and 6–11 y (22.2% and 8.2%), respectively. Milk was the leading source of calcium (22.7%) in 12–18-y olds. Milk and flavored milk were the first and second food sources of vitamin D in 2–5-y olds (55.7% and 12%) and 6–11-y olds (46.7% and 15.2%, respectively). Milk was the leading source of vitamin D (51.3%) in 12–18-y olds. Milk was the leading source of potassium in 2–5-y olds (21.1%) and 12–18-y olds (12.4%), respectively; milk and flavored milk were the first and third leading sources of potassium (15.6% and 5.7%, respectively) in 6–11-y olds. Milk and flavored milk were not leading contributors of % added sugars. Milk was the first food source of SFA (18.4%) in 2–5-y olds; second in 6–11-y olds (10.1%); and second for 12–18-y olds (10%)	Leading source of saturated fat in the diet	Leading source of 3 of 4 nutrients of public health concern in the diet
Fayet-Moore et al. 2019 [13]	Assess associations among milk drinking and milk, dairy, and micronutrients intakes compared with milk consumption from other sources or milk avoidance among children and adolescents	Cross-sectional secondary analysis of 2011–2012 Australian National Nutrition and Physical Activity Survey	*n =* 2812 Australian children and adolescents 2–18 y of age	Flavored and plain milk drinkers had higher total daily milk (480 g; 95% CI: 459, 501 g and 445 g, 95% CI: 427, 462 g, respectively) and calcium (1049 ± 18 mg and 980 ± 15 mg, respectively) intakes than all other groups. Plain and flavored milk drinkers and nondrinkers of milk had the lowest prevalence of SSB intake (*P <* 0.001)	ND	Higher micronutrient intake compared with milk nondrinkers Lower SSB intake compared with milk nondrinkers
Leme et al. 2019 [20]	Identify most commonly consumed foods by adolescents contributing to percentage of total energy, added sugars, SFA, sodium and total gram intake per day	Cross-sectional analysis of data from NHANES 2011–2014	*n =* 3156 adolescents 10–19 y of age	Leading sources of total energy were SSB (7.8%); sweet bakery products (6.9%); and mixed dishes-pizza (6.6%). Highest food sources of total gram amount consumed were plain water (33.1%); SSB (15.8%); milk (7.2%). Three highest food sources of added sugars were SSB (42.1%); sweet bakery products (12.1%); and coffee and tea (7.6%)	ND	ND
Kassis et al. 2022 [21]	Estimate the usual intakes of fiber, iron, zinc, calcium, folate, vitamin D, and vitamin A and the top foods that contribute to them among children in the UAE	Cross-sectional analysis	*n =* 1102 children 6–11.9 y of age	Main contributors to iron intake were infant/young child formula and infant cereal in children under 4 y, and fortified grains and meat/fish for children over 4 y. Vitamin D was inadequate across all age groups. Top sources of vitamin D were fortified milks	ND	Source of nutrients of concern
Gopinath, et al. 2014 [22]	Assess dairy intake and determine predictors of adequate dairy consumption during adolescence	Prospective analysis from semiquantitative FFQ	*n =* 634 Australian children and adolescents 12–17 y of age	Mean total dairy intake decreased from 1.62 servings per day at age 12 to 1.40 servings per day at age 17 (*P <* 0.0001). Consumption of ≥3 total dairy servings per day decreased from 8.5% of 12-y olds to 6.2% of 17-y olds (*P <* 0.005). Participants whose parents had higher education were 85% more likely to have higher than median intakes of dairy during the 5 y (OR = 1.85; 95% CI: 1.18, 2.91). ≥2 servings/wk of flavored milk consumption was associated with ∼5 fold greater likelihood of maintaining intakes of dairy foods above the median during adolescence	ND	Increased likelihood of maintaining dairy consumption during adolescence
Quann and Adams, 2013 [23]	Quantify the impact of changes in flavored milk availability on school children’s milk consumption	Intervention	*n =* 49 elementary schools	Removal of flavored milk on 1 to all days of the week was associated with 26% reduction in milk sales and 11.4% increase in the percentage of milk discarded, resulting in and estimated 37.4% decrease in milk consumption	ND	Increase milk consumption
Thompson et al. 2020 [24]	Examine the effect of policy that removed chocolate milk from secondary schools on students’ milk consumption and estimated milk-related nutrient intake	Intervention	*n =* 3158 secondary school students in 6th–12th grade, pre-policy *n =* 2966 students secondary school students in 6th–12th grade, post-policy	Proportion of students selecting milk declined 13.6% from 89.5% pre-policy to 75.9% post-policy (95% CI: 10.8%, 16.4%, *P <* 0.05). Proportion of milk wasted remained stable with 37.1% pre-policy to 39.3% post-policy (95% CI: −0.2%, 4.6%, *P <* 0.05). Average per-student milk consumption declined by 1oz per student from 4.8 oz pre-policy to 3.8 oz post-policy (95% CI: −1.1, −0.7 oz, *P <* 0.05). NS reductions observed for intake of calcium, protein, or vitamin D from milk. Estimated added sugars from milk declined by 3.1 g per student (95% CI: −3.2 g, −2.9 g, *P <* 0.05)	Increased intake of added sugars	Increased consumption of milk

Abbreviations: EAR, estimated average requirement; HEI, Healthy Eating Index; PA, physical activity; RTEC, ready-to-eat cereals; SSB, sugar-sweetened beverage; WC, waist circumference.

Cross-sectional analyses of nationally representative survey data help assess the role of flavored milk in diet quality among children and adolescents. NHANES 1999–2002 data among 7557 children and adolescents ages 2–18 y found that flavored milk consumption was associated with higher total milk intake, and higher intake of vitamin A, calcium, phosphorus, magnesium, and potassium compared with milk nondrinkers [5]. Whereas saturated fat consumption was higher in milk and flavored milk drinkers than in milk nondrinkers, added sugar was not [5]. A cross-sectional analysis from the 2007 Australian National Children’s Nutrition and Physical Activity Survey reported similar findings in a sample of 4487 Australian children and adolescents, but also compared flavored milk drinkers with plain milk drinkers and reported that flavored milk drinkers were more likely to meet the estimated average requirement (EAR) for calcium than exclusive plain milk drinkers, with 9–16-y olds being 1.7 times more likely to meet the EAR for calcium [12]. Flavored milk drinkers also had higher intakes of total sugar and energy than exclusive plain milk drinkers, but there were no differences in anthropometric measures between groups [12]. Similar findings were reported from NHANES 2001–2012 data in which flavored milk consumers had a higher percentage of meeting the EAR for calcium [6]. In children and adolescents age 14–18-y old, flavored milk consumers consumed higher percentage of energy from saturated fat than flavored milk nonconsumers; they also consumed significantly higher calcium and vitamin D, helping to close the gap in consumption of nutrients of public health concern [6]. Using the United States Department of Agriculture’s Food Data Central’s legacy foods dataset, one cup of flavored milk typically served in schools contains ∼12 g of naturally occurring lactose, and an average of 8 g of added sugars (Table 3). This is due in large part to recent innovations that have been focused on reducing the amount of both total calories and added sugars in flavored milk. In fact, since 2007, these innovations have led to a 50% reduction of added sugars in flavored milk served in schools without the use of artificial and non-nutritive sweeteners [25]. The standard cup of flavored milk that is currently served in schools contains ∼8.2 g of added sugars and contains 126 calories, just 29 calories more than plain white milk [25]. Overall, flavored milk contains less added sugar than other beverages consumed by children, such as fruit drinks and soft drinks (see Table 3).

**TABLE 3:** Nutrient comparison of milk, plant alternatives, and sugar-sweetened beverages

Per 8 oz serving	Plain, unsweetened, unflavored				Sugar-sweetened beverages					Flavored
	Dairy milk (1%)	Dairy milk (nonfat)	Soy	Almond	Fruit juice	SSB/soda	Oat	Soy	Almond	Dairy milk
Calories	102	83	80	37	117	101	140	150	73	143
Protein (g)	8	8	7	1	2	0.22	3	9	1	8
Fat (g)	2	0.2	4	2	0.3	0	7	5	2	2
Total CHO (g)	13	13	4	3	21	26	16	19	13	20
Added sugar (g)	0	0	0	0	0	26	7	14	10	8
Calcium (mg)	305	299	299	449	349	5	350	470	432	402
Vitamin D (mcg)	3	3	0	2	0	0	4	3	2	6
Potassium (mg)	366	382	299	164	443	2	390	470	156	479
Vitamin B12 (mcg)	1	1	3	0	0	0	1	2.5	0	1
Vitamin A (mcg)	142	149	140	220	5	0	160	140	210	429
Riboflavin (mg)	0.5	0.5	0.5	0	0.1	0	1	0.44	0	1
Magnesium (mg)	27	27	39	15	27	2	—	40	15	60
Zinc (mg)	1	1	1	0.2	0.2	0.02	—	—	0.2	3
Selenium (mcg)	8	8	6	0.2	0.2	0	—	—	0.2	9
Choline (mg)	43	38	—	8	15	1	—	—	7	44

SSB, sugar-sweetened beverage.

—Blank cells indicate data not available.

Dairy milk (1%); USDA ARS FoodData Central, milk, low-fat, fluid, 1% milk fat, with added vitamin A and vitamin D (SR Legacy, 170872).

Dairy milk (nonfat); USDA ARS FoodData Central, milk, nonfat, fluid, with added vitamin A and vitamin D (fat free or skim) (SR Legacy, 171269).

Soy milk, unsweetened; Product: USDA: Silk; Supplier: USDA SR-28.

Almond milk, Silk Pure Almond, Unsweetened; Product: FNDDS; Supplier: USDA ARS FNDDS.

Fruit juice; USDA ARS FoodData Central, orange juice, chilled, includes from concentrate, with added calcium (SR Legacy, 169920).

SSB/soda; USDA ARS FoodData Central, beverages, carbonated, lemon-lime soda, no caffeine (SR Legacy, 173205).

Oat milk, Barista Edition; Product: Oatly: Supplier: Oatly.

Soy; silk chocolate soymilk; https://silk.com/plant-based-products/soymilk/chocolate-soymilk/?gclid=EAIaIQobChMIq57diNje_gIVEx19Ch0FlQvAEAAYASAAEgKGRvD_BwE&gclsrc=aw.ds

Almond milk; Product: FNDDS; Supplier: USDA ARS FNDDS.

Dairy milk; USDA ARS FoodData Central, beverages, chocolate malt powder, prepared with 1% milk, fortified (SR Legacy, 171874).

A cross-sectional analysis of NHANES 2003–2006 data quantified contributions from flavored milk and reported that flavored milk contributed up to 6% total energy, 12% saturated fat, 3% sodium, and 4% added sugars to the diets of children and adolescents 2–18 y of age; flavored milk also contributed 5% vitamin A, 11% vitamin D, 6% calcium, 4% potassium, 3% magnesium, and 4% phosphorus [15]. The results of these cross-sectional analyses indicated that whereas flavored milk contributed a modest amount of saturated fat and energy to the diets of children and adolescents, it also helped to close gaps and meet recommendations for total dairy and micronutrient intakes, particularly nutrients of public health concern, including calcium, vitamin D, and potassium.

Current data support that flavored milk helps drive total milk consumption, and the removal of flavored milk from the diets of children and adolescents may result in unintended consequences. In a prospective analysis of 634 Australian adolescents aged 12–17, researchers observed a decline in total dairy consumption and found that the consumption of 2 servings or more of flavored milk per week was associated with an ∼5-fold greater likelihood of maintaining intakes of dairy foods above the median during adolescence [22]. Concerns over childhood obesity have led to substantial reformation of school nutrition policies in America over the last 2 decades [18]. A shift toward the replacement of SSB with water, plain milk, and lower-fat flavored milks has helped decrease the availability of nutrient-poor foods and beverages in schools [18], but flavored milks continue to be scrutinized by some, for their added sugar and energy contents [26]. In the United States, it has been demonstrated that the removal of flavored milk from schools results in a decline of total milk consumption [[23], [24], [27]]. In a study looking at the removal of flavored milk between the 2010–2011 and 2011–2012 school years, average daily participation in the National School Lunch Program decreased by 6.8% and daily milk sales decreased by nearly 11% [27]. In elementary schools, the removal of flavored milk on one to all days of the week was associated with a 26% reduction in milk sales and an 11.4% increase in the percentage of milk discarded, resulting in an estimated 37.4% decrease in milk consumption [23]. In secondary schools, the proportion of students selecting milk with their meals declined 13.6%, resulting in estimated one ounce decline in milk consumption from 4.8 to 3.8 oz per student when flavored milk was removed from schools [24]. This decline was not associated with significant reductions to calcium, protein, or vitamin D provided by milk, but was associated with a decline in estimated added sugars from milk by 3.1 g per student, prompting researchers to recommend the removal of flavored milk from secondary schools [24]. Considerations with this study include a lack of assessment of nutrient composition of the school lunch overall, the students’ daily nutrient intake, and the nutrient intake of the 13.6% of students who did not select milk with lunch. In addition, these findings do not account for the potential impact of habituation to the removal of flavored milk and resulting behavior modification over the long term. Availability of these data would help put these changes in nutrient intakes from milk into a broader context of the total diet where they may, or may not have a significant impact. For example, a cross-sectional analysis of data from NHANES 2011–2014 indicated that among a sample of 3156 adolescents aged 10–19 y, flavored milk was not a leading source of added sugars in the diet [20].

## The Role of Flavored Milk in Appetite, Food Intake, Body Weight, and Body Composition among Children and Adolescents

To date, there is insufficient evidence to support a link between flavored milk consumption and childhood or adolescent obesity or its inclusion in policy aimed at reducing the consumption of SSB, such as colas or fruit drinks. Childhood and adolescent obesity is a global epidemic that has risen 10-fold over the past half-century [28]. Because of its potential to contribute energy and added sugar to the diet, the role of flavored milk in childhood and adolescent obesity has been questioned [26]. Unlike colas or fruit drinks, flavored milk is nutrient dense and contributes key shortfall nutrients including calcium, vitamin D, and potassium to the diets of children and adolescents [1]. The moderate consumption of flavored milk is positively associated with nutrient intakes and not with adverse effects on weight status in United States children and adolescents [5].

Systematic reviews aimed at examining the relationship between beverage consumption and body composition did not implicate flavored milk consumption as a risk factor for overweight or obesity (Table 4) [29,30]. A systematic review conducted for the 2020 Dietary Guidelines Advisory Committee indicated that there was limited evidence that milk consumption was not associated with adiposity in children and that there was insufficient evidence about the relationship between types of milk, be it by fat or by flavor, and adiposity in children [30]. A more recent systematic review and meta-analysis of randomized controlled trials indicated that the substitution of flavored milk or noncaloric beverages for SSB did not change BMI *z*-score; however, it did reduce body fat percentage by up to 30% in children and adolescents [29]. Although a direct causal relationship was not established between flavored milk intake and decreased body fat, flavored milk was likewise not implicated as a risk factor for overweight or obesity. On the basis of the available literature (see Table 4), there does not appear to be an association between flavored milk consumption and obesity. However, the evidence is limited and additional research is warranted.

**TABLE 4:** Studies assessing the role of flavored milk in child and adolescent body composition and metabolic health

Reference	Objective(s)	Design	Sample	Relevant results	Cost of flavored milk consumption	Benefit of flavored milk consumption
Murphy et al. 2008 [5]	Compare nutrients intakes and body measure among children and adolescents drinking flavored milk (with or without plain milk), exclusively plain milk, and no milk	Cross-sectional analysis of NHANES 1999–2002	*n =* 7557 children and adolescents 2–18 y of age	Flavored milk intake was associated with higher total milk intake (*P <* 0.05). Vitamin A, calcium, phosphorus, magnesium, potassium, and saturated fat (adjusted for energy intake and age) were comparable among milk drinking groups; higher than milk nondrinkers (*P <* 0.05). Added sugars intake did not differ between flavored milk drinkers and milk nondrinkers. BMI of milk drinkers was comparable with or lower than milk nondrinkers (*P <* 0.05)	ND	Higher nutrient intake and comparable added sugars and BMI than milk nondrinkers
Fayet et al. 2013 [12]	Determine associations among milk drinking (plain or flavored) and milk and nutrient intakes in children	Cross-sectional analysis of 2007 Australian National Children’s Nutrition and Physical Activity Survey	*n =* 4487 Australian children and adolescents 2–16 y of age	Milk drinking (plain or flavored) was associated with higher total milk, calcium, phosphorus, magnesium, potassium, and iodine intake in and were more likely to meet the EAR for calcium in comparison with milk nondrinkers (*P <* 0.05). Flavored milk drinkers were more likely to meet the EAR for calcium than exclusive plain milk drinkers, with 9–16-y olds being 1.7 times more likely to meet the EAR for calcium than exclusive plain milk drinkers (*P <* 0.01). Flavored milk drinkers had higher intakes of total sugar and energy than exclusive plain milk drinkers (*P <* 0.05). NS difference between BMI, WC or PA levels between flavored and exclusive plain milk drinkers	Higher total sugar and energy intake	Higher micronutrient intake compared with white milk drinkers and milk nondrinkers Greater likelihood of meeting calcium recommendations compared with white milk drinkers and milk nondrinkers No impact on body weight or composition despite greater sugar and energy intake than white milk drinkers
Brindal et al. 2013 [31]	Assess the differential effects of beverages varying in glycemic load and dairy composition on appetite, energy intake and cognitive function	Double-blind, randomized, crossover trial in which children consumed 1100 kJ of glucose beverage, milk beverage, or 50:50 glucose/milk beverage	*n =* 40 children 10–12 y of age	Blood glucose AUC values were different between drinks (*P =* 0.001), but did not sustain above the baseline for 3 h for any drink. NS effect of beverage on subjective appetite or energy intake. Girls showed improved short-term memory recall after consumption of either chocolate milk beverage (*P =* 0.014)	ND	Improved short-term memory recall in girls compared with glucose beverage
Mayer-Davis et al. 2020 [30]	Examine the relationship between beverage consumption and growth, size, body composition, and risk of overweight and obesity	Systematic review of literature found through PubMed, Cochrane, and Embase	Varied. Relevant results based on studies conducted in children and adolescents 2–18 y of age	Limited evidence that milk intake is not associated with adiposity in children. Insufficient evidence about the relationship between type of milk (that is, fat content, flavor) and adiposity in children. Limited evidence that higher milk intake is associated with greater increase in height compared with lower intake in children	ND	ND
Jakobsen et al. 2023 [29]	Investigate if an independent effect exists between foods, beverages or the composition of macronutrients and body composition in children and adolescents	Systematic review and meta-analysis of randomized controlled trials	*n =* 13 RCTs involving 1773 children and adolescents 4–18 y of age	Based on data from 3 studies, substituting SSB with noncaloric beverages or flavored milk reduced body fat percentage (−0.70; 95% CI: −0.78, −0.62; *P <* 0.001), but did not change BMI *z*-score	ND	Flavored milk reduced body fat percentage when substituted for SSB

Abbreviations: EAR, estimated average requirement; PA, physical activity; WC, waist circumference; RCT, randomized clinical trial; SSB, sugar-sweetened beverage.

There have been a few clinical trials that have assessed the role of flavored milk on appetite and food intake among children and adolescents (Table 5) [[31], [32], [33], [34]]. These trials have shown that flavored milk consumed either before or at mealtimes leads to a reduction in total energy intake during that meal, or a subsequent meal, as compared with water or isocaloric amounts of other beverages [28,30,32], although the mechanism of this outcome has yet to be fully explored in children. However, less is known about whether these benefits seen acutely would also be seen with longer-term/chronic consumption of flavored milk. This is a current gap in the literature that, if addressed, may help to elucidate this potential impact on appetite and energy intake as one of the mechanisms explaining why the published observational data support no significant difference among flavored milk consumers and nonconsumers in BMI, body weight, and measures of adiposity. To date, the data available do not suggest that flavored milk contributes to detrimental effects on childhood or adolescent energy intake or body composition.

**TABLE 5:** Studies assessing the role of flavored milk in child and adolescent appetite and food intake

Reference	Objective(s)	Design	Sample	Relevant results	Cost of flavored milk consumption	Benefit of flavored milk consumptions
Brindal et al. 2013 [31]	Assess the differential effects of beverages varying in glycemic load and dairy composition on appetite, energy intake and cognitive function	Double-blind, randomized, crossover trial in which children consumed 1100 kJ of glucose beverage, milk beverage, or 50:50 glucose/milk beverage	*n =* 40 children 10–12 y of age	Blood glucose AUC values were different between drinks (*P =* 0.001), but did not sustain above the baseline for 3 h for any drink. NS effect of beverage on subjective appetite or energy intake. Girls showed improved short-term memory recall after consumption of either chocolate milk beverage (*P =* 0.014)	ND	Improved short-term memory recall in girls compared with glucose beverage
Vien et al. 2017 [34]	Experiment #1: compare the effects of water and isocaloric amounts of 2% milk, chocolate milk, yogurt drink, and fruit punch on subjective appetite and food intake Experiment #2: Compare the effects of isocaloric amounts of 2% milk and fruit punch on subjective appetite, food intake, and glycemic and appetite hormone responses	Clinical trial	Experiment #1: *n =* 32 children and adolescents 9–14 y of age Experiment #2: *n =* 20 children and adolescents 9–14 y of age	Experiment 1: meal food intake was lower by 14% and 10%, respectively, after chocolate milk and yogurt drink (*P <* 0.001), but not milk, compared with water Experiment 2: milk led to higher pre-meal glucagon-like peptide-1 and post meal PYY than fruit punch (*P <* 0.01). NS in insulin between treatments	ND	Increased satiety Decreased short-term food intake
Bennett et al. 2018 [32]	Compare the effect of pre-meal consumption of commercially available SSB on subjective appetite and short-term food intake	Randomized crossover trial in which girls consumed 350 mL of fruit drink (154 kcal), cola (158 kcal), 1% chocolate milk (224 kcal), or water (control; 0 kcal)	*n =* 28 girls 9–14 y of age	Subjective appetite and thirst decrease after all beverages, but did not differ among beverages. Short-term food intake was suppressed after chocolate milk (15%; *P <* 0.001) and cola (11%; *P =* 0.02) compared with water control. Cumulative energy intake was not affected by beverage type	ND	ND
Poirier et al. 2019 [33]	Determine the effects of isovolumetric preloads of fruit-flavored drink, cola, 1% chocolate milk, and water on subjective appetite and food intake in boys	Clinical trial	*n =* 32 boys ages 11.8 ± 0.3 y of age	Food intake was reduced after cola (940 ± 46 kcal) and chocolate milk (878 ± 41 kcal) compared with water control (1048 ± 35 kcal) and after chocolate milk compared with the fruit drink (1005 ± 44 kcal) (*P <* 0.001). Average appetite was not affected by the treatment	ND	Decreased short-term food intake

Abbreviation: PYY, peptide YY.

## The role of flavored milk in risk of dental carries among children and adolescents

One concern related to flavored milk consumption and child and adolescent health is risk of increased dental carries associated with added sugars in the diet. The WHO recommends that free sugars be limited to <5% total energy to reduce risk of dental caries [35]. A cross-sectional study conducted in Canadian preschoolers that utilized FFQs submitted by children’s parents indicated that flavored milk was a leading contributor of free sugars to the children’s diets; it contributed 3.1% of total energy [36], which fell within recommendations from the WHO [35]. Another cross-sectional analysis conducted in Egyptian children ages 5–10 y that aimed to assess relationships between dietary habits, overweight and obesity, and dental carries, also by utilizing FFQs submitted by children’s parents, indicated an association between sweetened milk consumption and dental carries [37]. Unfortunately, the statistical analysis did not include adjustments for lifestyle factors, only for age and gender, making it difficult to determine the strength of the observed relationship. In a prospective analysis conducted in Brazilian children, an association between sweetened milk consumption between the ages of 10 and 11 y was associated with dental carries at 12–13 y of age [38]. The “sweetened milk and powdered chocolate” category in the study was comprised of products that contained upwards of 15 g of added sugar [38], nearly double that of flavored milk included in current United States school lunch offerings [25]. Research investigating the association between flavored milk consumption in the context of overall healthy dietary patterns and risk for dental carries is warranted.

## Discussion

In the United States, the triple burden of malnutrition, which includes undernutrition, nutrient inadequacies, and overweight or obesity, is a leading contributor to overall poor health. Diets that are energy dense and nutrient poor are key drivers of increased likelihood of childhood obesity, suboptimal growth and development, as well as increased risk of chronic diseases such as cardiovascular disease later in life. The reason milk remains an underpinning of dietary recommendations both in the United States and worldwide [39] is because of its unique nutrient package that provides shortfall nutrients, especially to the diets of children and adolescents. In the United States, milk and dairy foods provide high-quality protein and 3 of 4 nutrients of public health concern—calcium, vitamin D, and potassium—to the diets of children and adolescents [16]. Flavored milk is preferred and consumed by children worldwide and contributes to total dairy and micronutrient intakes, also closing gaps in nutrients of public health concern [26,39]. Children and adolescents who consume flavored milk adhere to healthier overall dietary patterns [5,6]. An unintended consequence of removing flavored milk from schools is a reduction in total milk and micronutrient intakes by children and adolescents [10,15,23].The DGA defines nutrients to limit in the diet as total energy (calories), added sugars, and saturated fat [3]. This review of the literature found that flavored milk was not a primary source of these nutrients to limit. In addition, flavored milk is a leading source of several nutrients of public health concern in children and adolescents as well as a major contributor of other essential nutrients including vitamin A, magnesium, and phosphorous. When consumed in moderation, ∼1–2 cups per day, flavored milk was associated with improved diet quality and metabolic health and was not observed to increase risk of dental carries (see Table 5) [40,41]. This narrative review analysis, which looked at the health and nutrition benefits of including flavored milk in the diet of United States children and adolescents, indicated that there was not convincing evidence to suggest a removal of flavored milk based on its content of energy, fat, or added sugars. Rather it found that flavored milk had an important role to play in the overall healthy dietary patterns of children and adolescents. Furthermore, emerging evidence supports the link between better overall diet quality during childhood and the adolescent years with better diet quality during adulthood and better cardiometabolic health [42,43].

Although it must be acknowledged that total added sugar consumption exceeding the 10% limit set by the DGA and/or in the context of excessive caloric intake can be problematic in contributing to overweight and obesity, most of the available evidence suggests that there is no significant effect between the relatively small contribution of added sugars in flavored milk served in schools or consumed within a calorically appropriate diet and risk of obesity [4,5,[35], [36], [37], [38]]. Moreover, one should consider the potential impact of removing flavored milk from school lunch programs and/or the overall diet. Elimination of flavored milk from schools has been demonstrated to decrease milk consumption that nutritionally results in fewer calories and less added sugar, but also less protein, calcium, and vitamin D, nutrients essential to bone health in children and adolescents [23]. Furthermore, the replacement of those nutrients with other foods has been demonstrated to be costly and can result in adding more calories and fat to school meals than if flavored milk was available on the menu [23]. Minor changes or supplementations with current school lunch menu offerings would not replace the nutrients lost from eliminating flavored milk [23]. The implications for children and adolescents may be nutrient shortfalls in the short term and the potential for chronic conditions, such a lower peak bone mass in the long term. In addition, removal of flavored milk from school lunches has been shown to result in a decline in student participation in school lunch overall [27]. This can have a negative impact on their nutrient intakes and overall health because, according to the USDA Food and Nutrition Service, children who participated in school meals consumed more dairy milk, fruits, and vegetables, 3 of the 4 commonly underconsumed food groups in the DGA, than nonparticipants [44]. Furthermore, they consumed fewer desserts, snacks, and nonmilk beverages [44]. These unintended consequences need to be considered. On the basis of current research, there is no strong evidence-based rationale for discouraging flavored milk from the diet of United States children and adolescents. Therefore, flavored milk should continue to be offered as part of school meals and accepted as part of total dairy intakes to support achieving current DGA dairy recommendations to improve nutrient adequacy.

### Author contributions

The authors’ responsibilities were as follows—KR-J, MAP: were responsible for design, literature review, writing, and final content; and have read and approved the manuscript.

### Author disclosures

The authors are employed by National Dairy Council.

### Funding

No outside funding was obtained for this article. This study is supported by National Dairy Council.
